# Depression and anxiety in parents of children with intellectual and developmental disabilities: A systematic review and meta-analysis

**DOI:** 10.1371/journal.pone.0219888

**Published:** 2019-07-30

**Authors:** Nathaniel Scherer, Ibone Verhey, Hannah Kuper

**Affiliations:** 1 International Centre for Evidence in Disability, Department of Clinical Research, London School of Hygiene & Tropical Medicine, London, United Kingdom; 2 Centre for Global Mental Health, London School of Hygiene & Tropical Medicine and King’s Health Partners, London, United Kingdom; Universita degli Studi di Ferrara, ITALY

## Abstract

**Introduction:**

Although caring for a child with intellectual and developmental disabilities (IDD) can have positive outcomes, parents may be at a greater risk of depression and anxiety, due to a number of associated stressors, such as increased caregiver demands and financial strain. This systematic review updates previous data, exploring the relationship between parenting a child with IDD and parental depression and anxiety.

**Methods:**

Five electronic databases were searched for eligible English-language articles, published between January 2004 and July 2018. All epidemiological study designs were eligible, provided the level of depression and/or anxiety was compared between parents of children (aged <18) with and without IDD. No limit was placed on geographic location. The proportion of positive associations between parenting a child with IDD and depression/anxiety were disaggregated by disability type, geographic region, and sample size. The percentage of parents at risk of moderate depression or anxiety were calculated using recognised clinical cut-off scores for each screening tool. Meta-analyses, in which pooled effect sizes of elevated depression and anxiety symptoms were calculated, were conducted across two IDD conditions, autism and cerebral palsy.

**Results:**

Of the 5,839 unique records screened, 19 studies fulfilled the inclusion criteria. The majority of studies were conducted in high-income (n = 8, 42%) or upper-middle income countries (n = 10, 53%). Of the 19 studies, 69% focused on parents of children with cerebral palsy (n = 7, 37%) or autism (n = 6, 32%). Nearly all studies found a positive association between parenting a child with IDD and depression (n = 18, 95%) and anxiety (n = 9, 90%) symptoms. Factors associated with higher levels of depression symptoms amongst parents of children with IDD included disability severity (n = 8, 78%) and lower household income (n = 4, 80%). Approximately one third (31%) of parents of children with IDD reach the clinical cut-off score for moderate depression, compared with 7% of parents of children without IDD. 31% of parents of children with IDD reach the cut-off score for moderate anxiety, compared with 14% of parents of children without IDD. The meta-analyses demonstrated moderate effect sizes for elevated depression amongst parents of children with autism and cerebral palsy.

**Conclusions:**

Results indicate elevated levels of depressive symptoms amongst parents of children with IDD. Quality concerns amongst the existing literature support the need for further research, especially in low- and middle-income countries.

## Introduction

The term “developmental disabilities” covers a diverse range of cognitive and physical impairments, each of which emerge during early child development and remain present throughout a person’s lifetime [1]. Developmental disabilities are prevalent in 15% of children aged 3 to 17 [2], and can limit an individual’s ability to engage in everyday activities, as areas of communication, learning, and mobility are often diminished. Falling under this umbrella term is intellectual disability, a condition characterised by severe limitations in intellectual functioning and adaptive behaviour, impairing a person’s ability to learn, understand, and apply complex information and skills [3]. Intellectual disability often occurs in unison with other developmental disabilities and the term “intellectual and developmental disabilities” (IDD) is often used to denote the co-morbid demonstration of both conditions.

Raising a child with IDD can generate positive outcomes for a family, including improved family closeness, personal growth, and importantly, joy [4–6]. That being said, a number of chronic stressors are inherent in raising a child with IDD, such as child behavioural problems [7], high caregiver demands [8], stigma [9], and financial strain [10]. As a result of these stressors, parents of children with IDD may be more vulnerable to depression and anxiety [11].

Depression is associated with a range of negative effects, including poorer physical health [12], lack of self-care, and limited social functioning [13]. Of additional concern is the well-established link between parental depression and disengaged parenting behaviours [14–16]. Without these reciprocal interactions, children have few opportunities to develop appropriate social skills during an important period of cognitive growth. This issue is particularly pertinent when discussing children with IDD, who face developmental impairments, regardless of parental interaction. Acting as a negative environmental factor, parental depression is likely to exacerbate disability in a child with IDD.

In 2006, Singer synthesised data on depression in mothers of children with IDD [17]. He identified 18 studies, of which 55% demonstrated a positive association. Overall, the prevalence of depression in mothers of children with IDD was estimated to be 29%, compared with 19% in mothers of typically developing children.

It has been over 12 years since Singer’s publication and this review seeks to update his findings and expand them across three key facets. Firstly, this review includes anxiety as well as depression, as evidence demonstrates high levels of co-morbidity of these conditions [18]. As with depression, parental anxiety can have a negative impact on child development, often leading to child anxiety and depression [19]. Secondly, depression and anxiety are considered in fathers, as well as in mothers. Fathers play a prominent role in caregiving and evidence demonstrates the importance of father-child interactions in child development [20], a vital consideration when observing the diminished interactions of fathers with depression and/or anxiety. Thirdly, Singer’s review was limited to papers published in Northern America and this review includes a growing evidence base from low- and middle-income countries (LMICs). The prevalence of IDD is generally considered to be higher in LMICs, given a number of risk factors, such as perinatal malnutrition and infection [21].

Building upon Singer’s review and meta-analysis, this review has three main objectives: (1) to narratively synthesise evidence on depression and/or anxiety in parents of children with IDD; (2) to identify factors associated with depression and/or anxiety in parents of children with IDD; (3) to estimate pooled effect sizes for the association between parenting a child with IDD and depression and/or anxiety.

## Materials and methods

This systematic review has been conducted in accordance with the Preferred Reporting Items for Systematic Reviews and Meta-Analysis (PRISMA) guidelines (S1 Table) [22]. The protocol for this review has been registered in the PROSPERO International Prospective Register of Systematic Reviews (CRD42018089891).

### Search strategy

Articles were retrieved from five bibliographic databases in July 2018: MEDLINE, PsycINFO, EMBASE, Web of Science, and CINAHL. The search was limited to studies published between January 2004 and July 2018, as the review builds upon Singer’s, conducted on studies from 1984 to 2003. The review was limited to peer-reviewed articles published in the English language. No limitations were placed on type of setting or geographic location.

Search terms were split across four facets: (1) IDD; (2) child; (3) parent; (4) depression and anxiety. Each of the search terms were derived from existing literature and Singer’s review. An example search strategy is provided in S2 Table.

### Inclusion and exclusion criteria

Inclusion criteria were created using a PICOS (Population, Intervention, Comparison, Outcomes, and Study Type) formulation [23].

1. Population: parents of children with and without IDD, where the child is cared for at home and under 18 years of age;
2. Intervention (exposure): presence of IDD in at least one child of the parents under study;
3. Comparison: presence of depression and/or anxiety in parents of a child with IDD and parents of a typically developing child;
4. Outcomes: levels of depression and/or anxiety, measured via a standardised screening tool or clinical diagnostic interview;
5. Study type: all quantitative study designs.

Exclusion criteria included: (1) qualitative studies, case reports, and review articles; (2) non-English full-text articles.

### Study selection

The principal author (NS) screened titles, abstracts, and full-text articles against the eligibility criteria. 20% of the titles and abstracts were reviewed by a second, independent reviewer (IV). Discrepancies were discussed until a consensus was reached. The reference list of each included article was screened for further eligible studies.

### Data extraction

Data extracted from the eligible articles included: (1) Study details: country, setting, methodological approach; (2) Participant characteristics: e.g. child age, parent sex; (3) Outcome measures and diagnostic tools; (4) Results: associated variables and diagnostic data, often in the form of a mean measurement score.

Study outcomes were classified into three categories (positive, null, and negative), as based on statistical significance, and disaggregated across study characteristics, including disability type, region, and methodological quality (see below). Studies were deemed ‘positive’ if the level of depression and/or anxiety symptoms was significantly higher in parents of children with IDD, compared with the control group. Inverse associations, in which the control group exhibited significantly higher levels of depression and/or anxiety symptoms, were characterised as ‘negative’. If a study did not detect a statistically significant difference, it was characterised as ‘null’.

To help interpret the difference in scores between groups, we estimated the percentage of parents scoring above or below clinical cut-off scores for moderate depression or anxiety when self-report measures were used, as they demonstrate symptom severity, not a clinical diagnosis. Only data collected from self-report measures with a recognised clinical cut-off score were included in the calculations. Depression and anxiety scores were standardised using the z statistic, from which we could estimate the percentage of parents above and below the cut-off score, using a z distribution table. These results assume that the depression and anxiety scores in each of the parent groups are normally distributed.

### Risk of bias

Eligible articles were assessed for risk of bias using the critical appraisal tool adapted from Lund et al. (2016) (Table 1) [24].

**Table 1:** Risk of bias criteria.

**Assessment criteria by study design**
Q1. Study design and sampling method is appropriate to the study question
Q2. Adequate sample size (>100), or sample size calculations undertaken
Q3. Response rate reported and acceptable (>70%)
Q4. Disability/impairment measure is clearly defined and reliable
Q5. Depression and/or anxiety measure is clearly defined and reliable
Q6. Potential confounders are taken into account in analysis
Q7. Confidence intervals are presented
**Case control (additional criteria)**
Q8. Cases and controls are comparable
Q9. Cases and controls are clearly defined
**Cohort (additional criteria)**
Q10. Groups being studied are comparable at baseline
Q11. Losses to follow up are presented and acceptable

The risk of bias of each study was independently assessed by the two reviewers (NS and IV), each of whom evaluated the 11 items into trichotomous ratings: ‘low, ‘medium, and ‘high’ risk. An overall rating was subsequently calculated (Table 2). Disagreements in the ratings were discussed until a consensus was reached.

**Table 2:** Risk of bias ratings.

Risk of Bias	
**Low**	All or almost all of the above criteria were fulfilled, and those that were not fulfilled were thought unlikely to alter the conclusions of the study
**Medium**	Some of the above criteria were fulfilled, and those not fulfilled were thought unlikely to alter the conclusions of the study
**High**	Few or no criteria were fulfilled, and the conclusions of the study were thought likely or very likely to alter their inclusion

### Meta-analysis

The meta-analysis synthesises depression and anxiety outcome data across the disability types for which there was sufficient comparable data. Hedges’ g was used to compute the standardised mean difference (SMD) of the outcome scores between cases and controls [25]. A pooled weighted mean effect size of elevated symptoms of depression and anxiety was calculated and adjusted, in line with the procedures outlined by Hedges and Olkin (1985) [25]. A random effects model was used, as the true effect size may differ from study to study, given the sample of functionally independent studies [26]. For each set of analyses, a test for homogeneity was conducted using the *I*^*2*^ statistic, an estimate of total variance in effect sizes attributable to between-study variance. The statistic is represented as a percentage, and the cut-off points of 25%, 50%, and 75% represent low, medium, and high heterogeneity [27].

## Results

**Fig 1:**
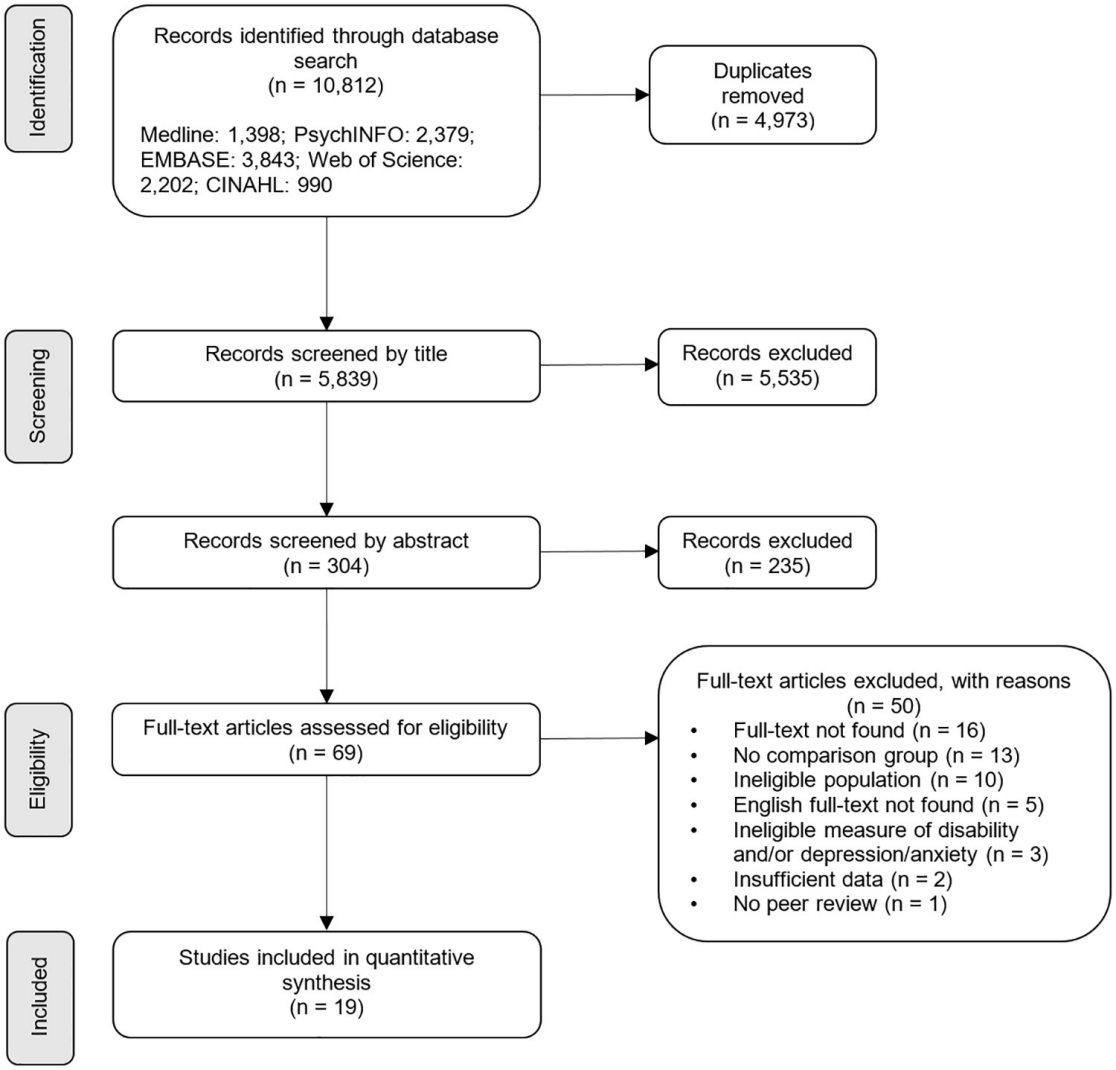
Study selection PRISMA flow diagram.

The database search generated 10,812 records, from which 4,973 duplicates were removed. 5,535 and 235 records were excluded during the screening of titles and abstracts, respectively. Of the 69 full texts assessed for inclusion, 19 were eligible (Fig 1). Screening the reference lists of eligible articles did not reveal further eligible studies.

### Study characteristics

The 19 studies included 3,303 parents of children with IDD and 9,519 parents of children without IDD, across 11 countries. Individual study characteristics are presented in Table 3.

**Table 3:** Key characteristics of included studies.

Source study	Country	Study design	Sample setting cases	Sample setting control	Sample size (% IDD)	IDD diagnostic method	Primary outcome	Risk of bias
**Autism**								
**Almansour, 2013** [28]	Saudi Arabia	Case-control	Hospital & support service	Hospital	100 (50%)	Clinical diagnosis	Depression& anxiety	Medium
**Gong, 2015** [29]	China	Case-control	Mental Health centre	School	302 (62%)	Clinical diagnosis	Depression & anxiety	Medium
**Ingersoll, 2010** [30]	USA	Case-control	Online portal	Online portal	165 (43%)	Parental report	Depression	Medium
**Lai, 2015** [31]	Singapore	Case-control	Specialised service	Student health centre	135 (54%)	Clinical diagnosis	Depression & anxiety	Medium
**Riahi, 2010** [32]	Iran	Case-control	Hospital	Hospital	61 (53%)	Clinical diagnosis	Depression & anxiety	High
**Yang, 2015** [33]	China	Case-control	Mental Health centre	School	1,682 (43%)	Clinical diagnosis	Depression	Medium
**Cerebral Palsy**								
**Altindag, 2007** [34]	Turkey	Case-control	Not reported	Not reported	119 (44%)	Clinical diagnosis	Depression & anxiety	Medium
**Basaran, 2013** [35]	Turkey	Case-control	Not reported	Not reported	203 (70%)	Clinical diagnosis	Depression & anxiety	Medium
**Cheshire, 2010** [36]	UK	Case-control	Specialised service	University advert	140 (50%)	Parental report	Depression & anxiety	Medium
**Kaya, 2010** [37]	Turkey	Case-control	Hospital	Hospital	141 (57%)	Clinical diagnosis	Depression	Medium
**Ones, 2005** [38]	Turkey	Case-control	Hospital	Hospital	92 (50%)	Clinical diagnosis	Depression & anxiety	Medium
**Unsal-Delialioglu, 2009** [39]	Turkey	Case-control	Hospital	Hospital	99 (49%)	Clinical diagnosis	Depression	Medium
**Yilmaz, 2013** [40]	Turkey	Case-control	Hospital	Hospital	230 (51%)	Clinical diagnosis	Depression & anxiety	Medium
**Multiple IDD: Combined**^a^								
**Cantwell, 2015** [41]	Republic of Ireland	Case-control	School & support service	University advert	173 (67%)	Parental report	Depression	Medium
**Norlin, 2013** [42]	Sweden	Case-control	Support service	Online portal	423 (25%)	Parental report	Depression	Medium
**Olsson, 2008** [43]	Sweden	Case-control	Support service	Online portal	430 (26%)	Parental report	Depression	Medium
**Multiple IDD: Disaggregated**^a^								
**Lach, 2009** [44]	Canada	Cross-sectional	Nationwide survey	Nationwide survey	8,400 (14%)	Clinical diagnosis	Depression	Medium
**Muammer, 2013** [45]	Turkey	Case-control	Specialised service	Not reported	76 (74%)	Clinical diagnosis	Depression	High
**Intellectual Disability**								
**Gogoi, 2017** [46]	India	Case-control	Mental Health centre	Regional survey	120 (50%)	Clinical diagnosis	Depression & anxiety	Medium

^a^ Studies in which multiple IDDs are represented in one sample. ‘Multiple IDD: Combined’ indicates that outcome data is combined across the sample. ‘Multiple IDD: Disaggregated’ indicates that the researchers have disaggregated outcome data by IDD type.

Almost all studies were conducted in either upper-middle income (n = 10, 53%) or high-income countries (n = 8, 42%), with a large number carried out in Europe (n = 11, 58%). Seven studies were undertaken in Turkey (n = 7), with the remainder in China (n = 2), Sweden (n = 2), Canada (n = 1), India, (n = 1), Iran (n = 1), Republic of Ireland (n = 1), Saudi Arabia (n = 1), Singapore (n = 1), UK (n = 1), and USA (n = 1).

The majority of studies focused on parents of children with cerebral palsy (n = 7, 37%) or autism (n = 6, 32%). Over a quarter of studies included multiple IDDs in one sample, either disaggregating results or combining them under a broader term (n = 5, 26%). Nearly all studies were case-control (n = 18, 95%), with the majority of cases and controls recruited through hospitals or specialised services (n = 9, 47% and n = 7, 37%, respectively). The majority of studies accommodated sample sizes of 100–200. All studies assessed depression, either in isolation (n = 9, 47%) or in tandem with anxiety (n = 10, 53%). When measuring depression, over half of the studies used the Beck Depression Inventory (BDI) or modified versions, such as the BDI-2r and BDI II (n = 10, 53%). All of the measurement tools, for both depression and anxiety, were self-report screening tools.

The mean ages of children ranged from 3.38 to 12.35, with very few adolescents included. Of the 19 studies, nine samples included mothers only. Of the remaining 10, the average percentage of mothers within all samples was 79%.

### Association between parenting a child with IDD and depression and anxiety

Outcome data, including standardised mean scores and standard deviations (disaggregated by fathers and mothers, where possible) and the measures of depression and anxiety, is provided in Tables 4 and 5. Table 6 presents a summary of key features.

**Table 4:** Percentage of parents of children with and without IDD scoring above clinical cut-off scores for moderate depression.

Source study	Outcome measure^a^	Mean score ± SD		P-value	Z Score		>Cut-Off (%)		Difference (%)
		With IDD	Without IDD		With IDD	Without IDD	With IDD	Without IDD	
**Autism**									
**Almansour, 2013**	HADS	9.5±3.2	5.0±3.0	<0.001	0.47	2	32%	2%	30%
**Gong, 2015**	SDS	**Fathers**: 45.3±9.4 **Mothers**:49.4±10.8	41.3±11.2	**Fathers**: <0.0001 **Mothers**: 0.006	**Fathers**: 1.56 **Mothers**: 0.98	1.67	**Fathers**: 6% **Mothers**: 16%	5%	**Fathers**: 1% **Mothers**: 11%
**Ingersoll, 2010**	CES-D	17.9±12.6	13.4±12.8	<0.05	0.17	0.52	44%	30%	14%
**Lai, 2015**^b^	DASS-D	3.2±3.7	2.4±3.1	<0.05	n/a	n/a	n/a	n/a	n/a
**Riahi, 2010**	GHQ	15.7±5.3	14.7±5.1	0.47	n/a	n/a	n/a	n/a	n/a
**Yang, 2015**	SDS	**Fathers**: 46.6±10.2 **Mothers**: 50.3±11.0	**Fathers**: 42.2±9.8 **Mothers**: 42.1±9.4	<0.0001	**Fathers**: 1.31 **Mothers**: 0.88	**Fathers**: 1.82 **Mothers**: 1.90	**Fathers**: 9% **Mothers**: 19%	**Fathers**: 4% **Mothers**: 3%	**Fathers**: 5% **Mothers**: 16%
**Cerebral Palsy**									
**Altindag, 2007**	BDI	18.8±8.6	9.1±4.8	<0.001	0.02	2.06	49%	2%	47%
**Basaran, 2013**	BDI	13.6±8.9	10.5±5.6	0.04	0.61	1.52	27%	6%	21%
**Cheshire, 2010**	HADS	7.0±3.9	3.9±3.3	<0.001	1.03	2.15	15%	2%	13%
**Kaya, 2010**	BDI	14.8±6.9	9.8±6.78	<0.0001	0.61	1.36	27%	9%	18%
**Ones, 2005**	BDI	18.3±10.3	7.3±7.5	<0.05	0.07	1.56	48%	6%	42%
**Unsal-Delialioglu, 2009**	BDI	19.7±12.0	13.2±7.8	0.002	-0.06	0.74	52%	23%	29%
**Yilmaz, 2013**	BDI	18.0±12.5	8.9±7.6	<0.001	0.08	1.33	47%	9%	38%
**Multiple IDD: Combined**									
**Cantwell, 2015**	HADS	8.7±4.1	5.1±3.7	<0.001	0.56	1.60	29%	6%	23%
**Norlin, 2013**^c^	BDI-2r	**Fathers**: -21.5±11.2 **Mothers**: -12.4±13.8	**Fathers**: -20.9±14.3 **Mothers**: -20.8±12.9	**Fathers**: >0.05 **Mothers**: <0.001	n/a	n/a	n/a	n/a	n/a
**Olsson, 2008**^d^	BDI-2r	**Fathers**: 105.4±10.9 **Mothers**: 96.3±13.9	**Fathers**: 107.3±12.4 **Mothers**: 104.8±12.8	<0.05	n/a	n/a	n/a	n/a	n/a
**Multiple IDD: Disaggregated**									
**Lach, 2009**^d^	CES-D	**Both**: 8.7±7.3 **Neuro**: 5.9±6.6	4.3±5.0	<0.0001	**Both**: 1.55 **Neuro**: 2.14	3.14	**Both**: 6% **Neuro**: 2%	0%	**Both**: 6% **Neuro**: 2%
**Muammer, 2013**^e^	BDI	MMG: 31.7±15.8 DG: 10.4±6.7 MG: 18±6.4	7.4±7.3	<0.001	**MMG**: -0.80 **DG**: 1.28 **MG**: 0.16	1.59	**MMG**: 79% **DG**: 10% **MG**: 44%	6%	**MMG**: 73% **DG**: 4% **MG**: 36%
**Intellectual Disability**									
**Gogoi, 2017**	BDI-II	21.9±6.1	6.7±5.3	<0.001	-0.31	2.51	62%	1%	61%

Abbreviations: HADS, Hospital Anxiety and Depression Scale; SDS, Zung Self-Rating Depression Scale; Center for Epidemiologic Studies Depression Scale; DASS-D, Depression and Anxiety Stress Scale—Depression; GHQ, General Health Questionnaire; BDI, Beck Depression Inventory

Percentages summary

**Overall**: with IDD = 31%; without IDD = 7%; difference = 24%

**Autism**: with IDD = 21%; without IDD = 9%; difference = 12%

**Cerebral palsy**: with IDD = 38%; without IDD = 8%; difference = 30%

**Multiple combined**: with IDD = 29%; without IDD = 3%; difference = 26%

**Multiple disaggregated**: with IDD = 28%; without IDD = 3%; difference = 15%

**Intellectual disability**: with IDD = 62%; without IDD = 1%; difference = 61%

^a^ HADS, cut-off score ≥11. SDS, cut-off score ≥60. CES-D, cut-off score ≥20. BDI, cut-off score ≥19. BDI-II, cut-off score ≥20

^b^ The z statistic and subsequent percentages were not calculated for measures with no recognised clinical cut-off score.

^c^ Norlin (2013) and Olsson (2008) have both used the BDI-2r, but they appear to have utilised a different scoring scale, hence the widely different scores across the two studies. Given this uncertainty, these studies were not included in the calculation of the z statistic.

^d^ Both: both a neurodevelopmental disorder and externalising behaviour problem; Neuro: neurodevelopmental disorder only

^e^ MMG: mental-motor disability group; DG: Down syndrome group; MG: mental disability group

**Table 5:** Percentage of parents of children with and without IDD scoring above clinical cut-off scores for moderate anxiety.

Source study	Outcome measure^a^	Mean score ± SD		P-value	Z Score		>Cut-Off (%)		Difference (%)
		With IDD	Without IDD		With IDD	Without IDD	With IDD	Without IDD	
**Autism**									
**Almansour, 2013**	HADS	10.5±3.9	5.6±3.8	<0.001	0.13	1.42	45%	8%	37%
**Gong, 2015**	SAS	**Fathers**: 38.8±8.2 **Mothers**: 42.0±9.4	36.4±8.1	**Fathers**: 0.041 **Mothers**: <0.0001	**Fathers**: 2.59 **Mothers**: 1.92	2.91	**Fathers**: 0.5% **Mothers**: 3%	0%	**Fathers**: 0.5% **Mothers**: 3%
**Lai, 2015**	DASS-A	2.7±3.3	2.6±3.6	<0.05	n/a	n/a	n/a	n/a	n/a
**Riahi, 2010**	GHQ	16.8±8.2	13.0±4.3	0.03	n/a	n/a	n/a	n/a	n/a
**Cerebral Palsy**									
**Altindag, 2007**	STAI 1–2	**STAI 1**: 71.9±5.7 **STAI 2**: 68.0±5.2	**STAI 1**: 32.4±2.3 **STAI 2**: 38.4±6.3	**STAI 1**: <0.001 **STAI 2**: <0.001	n/a	n/a	n/a	n/a	n/a
**Basaran, 2013**	BAI	14.2±11.3	9.0±5.9	<0.01	0.16	1.19	44%	12%	32%
**Cheshire, 2010**	HADS	9.3±4.5	7.1±3.9	0.002	0.38	1	35%	16%	19
**Ones, 2005**	BAI	10.8±9.0	9.8±8.8	0.91	0.58	0.71	28%	24%	4%
**Yilmaz, 2013**	BAI	20.1±15.5	9.7±8.0	<0.001	-0.27	0.79	60%	22%	38%
**Intellectual Disability**									
**Gogoi, 2017**	STAI	**SAI**: 47.6±5.3 **TAI**: 48.2±5.7	**SAI**: 35.1±5.8 **TAI**: 34.4±5.2	**SAI**: <0.001 **Tai**: <0.001	n/a	n/a	n/a	n/a	n/a

Abbreviations: HADS, Hospital Anxiety and Depression Scale; SAS, Zung Self-Rating Anxiety Scale; DASS-A, Depression and Anxiety Stress Scale—Anxiety; GHQ, General Health Questionnaire; STAI, State-Trait Anxiety Inventory; BAI, Beck Anxiety Inventory

Percentages summary

**Overall**: with IDD = 31%; without IDD = 14%; difference = 17%

**Autism**: with IDD = 16%; without IDD = 4%; difference = 12%

**Cerebral palsy**: with IDD = 42%; without IDD = 19%; difference = 23%

^a^ HADS = Hospital Anxiety and Depression Scale, cut-off score >11. SAS = Zung Self-Rating Anxiety Scale, cut-off score >60. BAI = Beck Anxiety Inventory, cut-off score >16.

**Table 6:** Summary of key characteristics of included studies.

		N	%
**Region**	Europe	11	58%
	North America	2	11%
	East Asia/Pacific	3	16%
	Middle East	2	11%
	South Asia	1	5%
**Country Income Status**	High	8	42%
	Upper-Middle	10	53%
	Lower-Middle	1	5%
**Disability Type**	Autism	6	32%
	Cerebral Palsy	7	37%
	Multiple IDD	5	26%
	Intellectual Disability	1	5%
**IDD Diagnosis**	Clinical diagnosis	14	74%
	Parental report	5	26%
**Outcome**	Depression	9	47%
	Anxiety	0	0%
	Both	10	53%
**Depression Measure**	Self-report screening tool	19	100%
	Structured interview screening tool	0	0%
	Diagnostic interview	0	0%
**Anxiety Measure**	Self-report screening tool	19	100%
	Diagnostic interview	0	0%
**Study Design**	Case-control	18	95%
	Cross-sectional	1	5%
**Sample Setting IDD**	Family support services	4	21%
	Hospital/specialised health service	9	47%
	Mental health centre	3	16%
	School	1	5%
	Online portal	1	5%
	Undisclosed	2	11%
	Other	1	5%
**Sample Setting Control**	Hospital/specialised health service	7	37%
	School	2	11%
	Undisclosed	3	16%
	Online portal	3	16%
	Other	4	21%
**Sample Size**	Smallest: 61	1	5%
	First quartile (25^th^ percentile): 100	5	26%
	Median (50^th^ percentile): 140	10	53%
	Third quartile (75^th^ percentile): 302	15	79%
	Largest: 8,400	19	100%
**Risk of Bias**	Low	0	0%
	Medium	17	89%
	High	2	11%

Nearly all studies reported a positive association between parenting a child with IDD and both depression (n = 18, 95%) and anxiety (n = 9, 90%) (S3 Table). With almost all studies demonstrating a positive association, it was not possible to identify those study characteristics that influenced the outcome (e.g. participant type, geographic location).

### Associated variables

Of the 19 studies assessing depression, 13 conducted multiple regression analysis, exploring factors that were associated with depression symptoms amongst case parents (S4 Table). Identified risk factors for parental depression symptoms included: relation to child (n = 4, 80% of studies in which this association was assessed found higher rates of depression in case mothers, compared with case fathers), disability severity (n = 8, 78%), and lower household income (n = 4, 80%). Only three studies assessed factors associated with anxiety, and the results do not demonstrate consistent patterns.

## Percentage of parents with elevated levels of depressive and anxiety symptoms

The percentage of parents with elevated levels of depressive symptoms is provided in Table 4. Table 4 shows that, on average, 31% of parents of children with IDD had elevated levels of depression (above the clinical cut-off for moderate depression), compared to 7% of parents of children without IDD—a 24% difference. Percentages for each disability type are shown in Table 4.

Similarly, the percentage of parents with elevated levels of anxiety symptoms is presented in Table 5. On average, 31% of parents of children with IDD had elevated levels of anxiety symptoms, compared to 14% of parents of children without IDD—a 17% difference. Percentages for each disability type are presented in Table 5.

### Quality appraisal and risk of bias

An overview of the methodological quality rating and risk of bias for each study is provided in S5 Table. All studies have received a minimum rating of ‘medium’ risk of bias, primarily because none of the studies adequately adjusted for confounders (e.g. through multivariate regression analysis). Similarly, no study provided confidence intervals for the means of the outcome measurement scores, although all studies provided the standard deviation. Other potential sources of bias include response rates, for which 68% of studies did not provide information or meet the appropriate cut-off. Half (53%) did not provide adequate information on the study design, including details of the sample setting. On a positive note, all studies used a measure of depression and/or anxiety that is clearly defined and reliable, with strong psychometric properties and extensive use in previous literature, although none utilise clinical diagnostic interviews, the gold standard assessment methodology. Of the 19 studies, two were deemed to have a ‘high’ risk of bias, and the remaining 17 a ‘medium’ risk of bias.

### Meta-analysis

Meta-analyses of the depression and anxiety scores were conducted across two disability types, autism and cerebral palsy, as they were deemed to be homogenous in study design.

**Fig 2:**
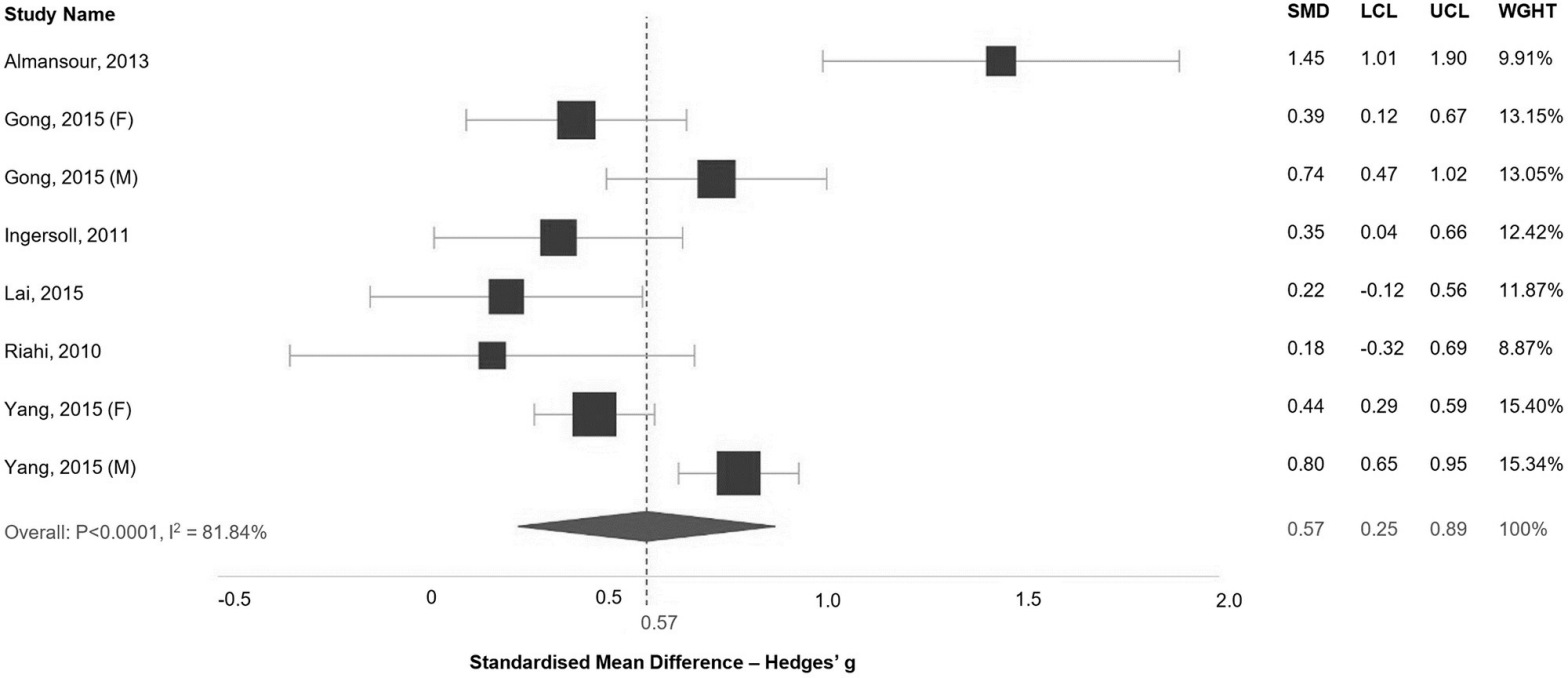
Standardised mean difference in depression scores between parents of children with autism and the control group. SMD: Standardised Mean Difference; LCL: Lower Confidence Interval; UCL: Upper Confidence Interval; WGHT: Weight.

Six studies focused on parents of children with autism were included within an analysis of the SMD in depression score between cases and controls (Fig 2). All studies demonstrated a positive association of elevated depression symptoms in parents of children with autism, although for Lai (2015) [31] and Riahi (2010) [32] the difference between cases and controls was not statistically significant. The pooled analysis demonstrates an effect size of elevated depression in parents of children with autism of 0.57 (95% CI: 0.25–0.89). There is, however, significant evidence of heterogeneity between study results (I^2^ = 81.84%, p < 0.0001). With regards to anxiety symptomology, four studies were included (S1 Fig). The effect size (0.54, 95% CI: -0.04–1.11) is not statistically significant.

**Fig 3:**
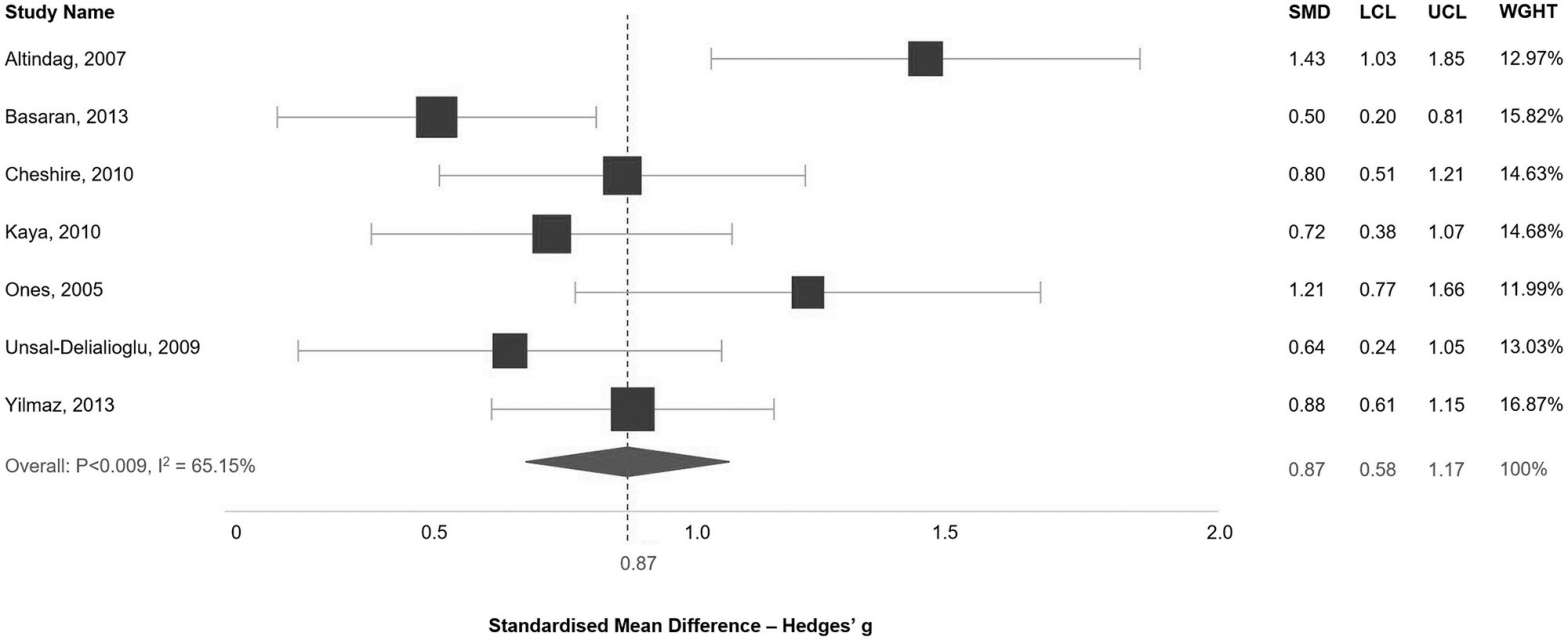
Standardised mean difference in depression scores between parents of children with cerebral palsy and the control group. SMD: Standardised Mean Difference; LCL: Lower Confidence Interval; UCL: Upper Confidence Interval; WGHT: Weight.

Seven studies focused on parents of children with cerebral palsy were included in the analysis of depression scores (Fig 3). The pooled analysis shows an effect size of elevated depression in parents of children with cerebral palsy of 0.88 (95% CI: 0.58–1.17). There is, however, strong evidence for a moderate level of heterogeneity (I^2^ = 65.15%, p = 0.009). Only four studies were included within an analysis of anxiety scores (S2 Fig), and again, the pooled estimate is not statistically significant (-0.46, 95% CI: -0.05–0.96).

## Discussion

This paper sought to update the findings of Singer (2006) [17], through a systematic review and meta-analysis, and identify whether parents of children with IDD are at a high risk of depression and anxiety. The review finds evidence for an association between parenting a child with IDD and depression and anxiety symptoms, with almost all studies reporting a positive relationship (95% and 90%, respectively). Findings are consistent across disability type, setting, and sample size. The percentage of parents with scores above the clinical cut-off for moderate depression were also higher in parents of children with IDD, although these estimates are based on a small number of studies and should be interpreted with caution. The pooled effect sizes demonstrate elevated depression in parents of children with autism and cerebral palsy, but not anxiety. The pooled effect sizes for elevated anxiety are not significant. Approximately one third (31%) of parents of children with IDD reach the clinical cut-off score for moderate depression, compared with 7% of parents of children without IDD. 31% of parents of children with IDD reach the cut-off score for moderate anxiety, compared with 14% of parents of children without IDD. The calculated percentages of parents with scores above the clinical cut-off are based on a small number of studies (especially with regards to anxiety), and results should be interpreted with caution.

In keeping with previous literature, four out of five studies (80%) reported higher levels of depression in case mothers, when compared with case fathers. This may result from higher caregiving responsibilities amongst mothers, or, alternatively, as a result of response bias, as men tend to underreport depressive symptoms when using self-report measures [47] (the methodology employed in all studies within this review). It is also important to note that case fathers exhibit higher levels of depression than control mothers and fathers (Table 4). Additional factors associated with depression include lower household income, consistent with the findings of Emerson (2006) [48], who demonstrated socio-economic position as accounting for 48–67% of the elevated risk for poor maternal well-being amongst mothers of children with intellectual disabilities. As a result of high caregiver demands, parents are often unable to work full-time, which, when combined with the cost of specialised services and medical bills, places families under serious financial strain [10]. Disability severity was associated with higher levels of parental depression symptoms, as increased severity may result in an array of behavioural problems and caregiver demands. Previous evidence suggests that the risk of depression is again elevated if the child has co-morbid disabilities, manifesting a mixture of physical and cognitive impairments [49–50].

The percentage of parents above the cut-off score for moderate depression and the results of the pooled meta-analyses of depression are consistent with that of Singer. It is interesting to note that the percentage of parents above the cut-off score (and the difference between groups) is higher than in Singer’s, despite us using the cut-off scores for moderate depression, as opposed to the cut-off for mild depression used by Singer. The largest effect size of elevated depression was found in studies focused on parents of children with cerebral palsy (Hedges’ g 0.88; 95% CI: 0.58–1.17), which likely results from the varied and physical demands placed on parents of children with cerebral palsy, as they deal with both physical and, in many cases, intellectual impairments. Although cerebral palsy can result in intellectual impairment, it is not always the case [51–52], and in this instance we can infer that inherent physical impairments are contributing to elevated depression amongst parents, with co-morbid cognitive impairments likely to compound this impact. Future studies may look to compare the levels of depression in parents of children with physical impairments only and those with both physical and intellectual (a comparison not explored by any study within this review). Neither of the meta-analyses of anxiety are statistically significant and it is difficult to draw strong conclusions. A larger number of studies available would have improved precision.

The findings of this review highlight several limitations within the available literature. That not one of the included studies adjusted their estimates for possible confounding variables is a major concern, and results may therefore lead to overestimated and erroneous conclusions [53]. Additionally, almost 70% of the 19 included studies focused on parents of children with autism and cerebral palsy, with just one study investigating parents of a child with intellectual disability. There are numerous other IDDs that have not been investigated, such as fragile X syndrome, spina bifida, and Down syndrome, and the omission of such IDDs has limited the generalisability of findings to a small coverage of disabilities. Interestingly, Singer’s review of the earlier published literature identified studies with a greater variety of IDDs, including Down syndrome (n = 3), spina bifida (n = 4), and intellectual disability (n = 5), as well as cerebral palsy and autism. Another important gap is the lack of evidence from LMICs, with no studies conducted in the continents of Africa or South America (although this may result from the exclusion of articles not published in the English language). However, compared to the earlier review by Singer, studies are included from Turkey, Saudi Arabia, China, Iran, and India, amongst others. Finally, we should note that all studies in this review utilise self-report measurement tools when assessing depression and anxiety, as opposed to the gold standard clinical diagnostic interview. In fact, shortly after Singer published his review, Bailey (2007) produced a critique of the literature on this topic [54], condemning an overreliance on these measurement tools, which are not designed for clinical diagnosis and can lead to overestimated conclusions [55]. Practicality often necessitates the use of these symptom screening tools and given their strong psychometric properties, we don’t consider their use a limitation in the context of this review, although we would prefer to see the gold standard methodology used in future studies, where possible.

### Strengths and limitations

Notably, this review includes a more thorough search strategy, with broader terminology and wider geographical reach, than that of Singer, who retrieved just 358 database results. The more detailed search parameters have yielded a higher number of database results, potentially providing a more comprehensive evidence composition. Adhering to PRISMA guidelines has ensured methodological rigour and transparency.

It is important to consider certain limitations when interpreting the findings of this review. Firstly, this review is limited to results published in the English language, and, although evidence suggests that such restrictions do not lead to systematic bias [56], the precision of pooled estimates, as well as the generalisability of results, are likely to have been improved if other languages had been included. Furthermore, the second reviewer was only able to assess 20% of the titles and abstracts during data extraction, although evidence suggests this methodology remains an effective procedure [57]. None of the meta-analyses included more than 10 studies, and tests for publication bias were therefore under-powered and not used. The cut-off scores to determine the percentages of parents with elevated depression and anxiety are only an estimate and the resultant percentages are based on heavy assumptions (e.g. normal distribution of symptoms), and so must be interpreted with caution.

Finally, the link between stress, anxiety, and depression, has not been analysed within this review, a potentially important omission, as parents of children with IDD often experience high levels of stress [58–59], which has been associated with the future occurrence of poorer physical [60] and mental health [61].

### Implications and recommendations

Parenting a child with IDD will often result in positive outcomes for parents and family members. It is important to recognise that the negative outcomes (depression and anxiety) discussed in this review are not inevitable. That being said, there is clearly a need for mental health support for parents of children with IDD, particularly for parents of children with autism and cerebral palsy. For instance, Peer and Hillman (2014) [62] encourage a focus on coping mechanisms, such as parental optimism and social support, to promote resilience, which may reduce the risk of depression and anxiety amongst parents of children with IDD. Further research is needed from low-income countries, reducing the reliance on data from high-income settings. Further research is also needed into the association with elevated anxiety symptoms and other types of IDD. Future research must minimise potential sources of bias and make certain that results are adjusted for potential confounding variables, ensuring true effect size estimates.

## Conclusions

The findings of this systematic review provide evidence that parenting a child with IDD is associated with elevated levels of depressive symptoms. Almost one third (31%) of parents of children with IDD in this review were estimated to experience moderate depression, 24% higher than the estimate for parents of children without IDD. Although further evidence is needed to improve the evidence base, there is an apparent need for specific services for these parents.

## Supporting information

S1 Table PRISMA checklist. (PDF)

S2 Table Search terms and example search strategy. (PDF)

S3 Table Association between parenting a child with IDD and depression and anxiety, by study characteristics. (PDF)

S4 Table Variables associated with depression and anxiety in parents of children with IDD. (PDF)

S5 Table Risk of bias, by IDD. (PDF)

S1 Fig Standardised mean difference in anxiety scores between parents of children with autism and the control group. (PDF)

S2 Fig Standardised mean difference in anxiety scores between parents of children with cerebral palsy and the control group. (PDF)
