# Supplementary material for: Depression and anxiety in parents of children with intellectual and developmental disabilities: A systematic review and meta-analysis
Source: PLoS One. 2019 Jul 30;14(7):e0219888. doi: 10.1371/journal.pone.0219888 (PMC6667144; doi:10.1371/journal.pone.0219888)
Supplement: S2 Table — (PDF) [file pone.0219888.s002.pdf]

S1 Table 1. Database search terms

Category	Search Terms
**IDD**	"intellectual disability", "intellectual impairment", "learning disability", "developmental disability", "mental retardation", "learning difficulty", "mental deficiency", "intellectual deficiency", "fragile X syndrome", "Down syndrome", "fetal alcohol syndrome", "autism", "Asperger syndrome", "Rett syndrome", "childhood disintegrative disorder", "Heller's syndrome", "pervasive developmental disorder" "cerebral palsy", "traumatic brain injury", "spina bifida"
**Child**	"child", "kid", "adolescent", "teenager", "youth"
**Parent**	"mother", "father", "carer", "caregiver", "parent", "family"
**Depression and Anxiety**	"depression", "anxiety", "depressive", "depressive disorder", "depressive symptom", "mood disorder", "affective disorder", "unipolar depression", "generalised anxiety disorder", "anxiety disorder"

S1 Table 2. Full MEDLINE database search strategy

1	(intellectual* dis* or intellectual* impair* or learning dis* or developmental dis* or mental retardation or learning difficult* or mental deficiency or intellectual* deficien* or fragile X syndrome or down* syndrome or f?etal alcohol syndrome or autism or autistic or asperger* or Rett syndrome or childhood dis?integrative disorder or pervasive developmental disorder or Heller* syndrome or cerebral palsy or traumatic brain injury or spina bifida).mp. [mp=title, abstract, original title, name of substance word, subject heading word, floating sub-heading word, keyword heading word, protocol supplementary concept word, rare disease supplementary concept word, unique identifier, synonyms]
2	(child* or adolescent* or teenager* or youth*).mp. [mp=title, abstract, original title, name of substance word, subject heading word, floating sub-heading word, keyword heading word, protocol supplementary concept word, rare disease supplementary concept word, unique identifier, synonyms]
3	(mother* or father* or carer* or caregiver* or parent* or famil*)*).mp. [mp=title, abstract, original title, name of substance word, subject heading word, floating sub-heading word, keyword heading word, protocol supplementary concept word, rare disease supplementary concept word, unique identifier, synonyms]
4	(depression or depressed or anxiety or depressive or depressive disorder or depressive symptom* or mood disorder or affective disorder or uni?polar depression or generali?ed anxiety disorder or anxiety disorder or anxiety symptom*).mp. [mp=title, abstract, original title, name of substance word, subject heading word, floating sub-heading word, keyword heading word, protocol supplementary concept word, rare disease supplementary concept word, unique identifier, synonyms]
5	1 and 2 and 3 and 4
