# Supplementary material for: Depression and anxiety in parents of children with intellectual and developmental disabilities: A systematic review and meta-analysis
Source: PLoS One. 2019 Jul 30;14(7):e0219888. doi: 10.1371/journal.pone.0219888 (PMC6667144; doi:10.1371/journal.pone.0219888)
Supplement: S3 Table — (PDF) [file pone.0219888.s003.pdf]

S2 Table. Association between parenting a child with IDD and depression and anxiety, by study characteristics

		Association of caring for a child with IDD and depression/anxiety					
		Depression			Anxiety		
		Positive	Negative	Null	Positive	Negative	Null
**Overall**		95%	0%	5%	90%	0%	10%
**Disability Type**	Autism	83%	0%	17%	100%	0%	0%
	CP	100%	0%	0%	80%	0%	20%
	Multiple	100%	0%	0%	0%	0%	0%
	ID	100%	0%	0%	100%	0%	0%
**Region**	Europe	100%	0%	0%	80%	0%	20%
	North America	100%	0%	0%	0%	0%	0%
	East Asia/Pacific	67%	0%	33%	50%	0%	50%
	Middle East	50%	0%	50%	100%	0%	0%
	South Asia	100%	0%	0%	100%	0%	0%
**Sample Size**	0-99	75%	0%	25%	50%	0%	50%
	100-199	100%	0%	0%	100%	0%	0%
	200-499	100%	0%	0%	100%	0%	0%
	>500	100%	0%	0%	0%	0%	0%
**Risk of Bias**	Low	0%	0%	0%	0%	0%	0%
	Medium	100%	0%	0%	89%	0%	11%
	High	50%	0%	50%	100%	0%	0%
