# Supplementary material for: Depression and anxiety in parents of children with intellectual and developmental disabilities: A systematic review and meta-analysis
Source: PLoS One. 2019 Jul 30;14(7):e0219888. doi: 10.1371/journal.pone.0219888 (PMC6667144; doi:10.1371/journal.pone.0219888)
Supplement: S4 Table — (PDF) [file pone.0219888.s004.pdf]

S3 Table. Factors associated with depression in parents of a child with IDD

Variables	Study	Association	P-value	% Positive
Characteristics of Child				
Age of child	Almansour, 2013	No association	0.54	0/4 (0%)
	Cantwell, 2015	No association	Not reported	
	Unsal-Delialioglu, 2009	No association	>0.05	
	Yang, 2016	No association	>0.05	
Disability severity	Altindag, 2007	Positive correlation with worse CP motor function	<0.001	7/9 (78%)
	Basaran, 2013	Positive correlation with worse CP motor function	<0.01	
	Gong, 2015	Positive correlation with autism severity	Not reported	
	Ingersoll, 2011	Positive correlation with autism severity	<0.01	
	Kaya, 2010	No correlation with CP motor function	>0.05	
	Ones, 2005	No correlation with CP motor function	0.52	
	Unsal-Delialioglu, 2009	Positive correlation with speech defects	0.036	
	Yilmaz, 2013	Positive correlation with worse CP motor function	<0.001	
	Yang, 2016	Positive correlation with autism severity	Not reported	
Time since diagnosis	Almansour, 2013	No association	0.19	0/1 (0%)
Characteristics of Parent				
Relation to child (i.e. mother or father)	Almansour, 2013	No association	0.99	4/5 (80%)
	Gong, 2015	Higher levels of maternal depression	<0.0001	
	Norlin, 2013	Higher levels of maternal depression	<0.01	
	Olsson, 2008	Higher levels of maternal depression	<0.05	
	Yang, 2016	Higher levels of maternal depression	<0.0001	
Parent age	Almansour, 2013	No association	0.53	0/3 (0%)
	Ingersoll, 2011	No association	>0.05	
	Yilmaz, 2013	No association	>0.05	

Variables	Study	Association	P-value	% Positive
Education level	Almansour, 2013	No association	0.07	2/6 (33%)
	Gong, 2015	Negative correlation with education level	<0.05	
	Ingersoll, 2011	No association	>0.05	
	Unsal-Delialioglu, 2009	No association	>0.05	
	Yang, 2016	Negative correlation with education level	<0.01	
	Yilmaz, 2013	No association	>0.05	
Number of children	Almansour, 2013	No association	0.49	0/3 (0%)
	Cantwell, 2015	No association	Not reported	
	Yilmaz, 2013	No association	>0.05	
Number of children with IDD	Almansour, 2013	Higher levels in cases with 3 or more autistic children	0.003	1/1 (100%)
Parenting stress	Gong, 2015	Positive correlation with parental stress	<0.01	3/3 (100%)
	Ingersoll, 2010	Positive correlation with parental stress	<0.05	
	Yang, 2016	Positive correlation with parental stress	<0.01	
Household income	Gong, 2015	Negative correlation with income level	<0.05	4/5 (80%)
	Norlin, 2013	Positive correlation with economic risk	<0.01	
	Unsal-Delialioglu, 2009	Negative correlation with income level	0.007	
	Yang, 2016	Negative correlation with income level	<0.01	
	Yilmaz, 2013	No association	>0.05	
Stigma	Cantwell, 2015	Positive correlation with stigma	0.04	1/1 (100%)
Marital quality	Norlin, 2013	Negative correlation with marital quality	<0.001	1/1 (100%)
Marital status	Ingersoll, 2010	Negative correlation with marital status (i.e. those married have reduced levels of depression)	<0.01	1/1 (100%)
