# Supplementary material for: Depression and anxiety in parents of children with intellectual and developmental disabilities: A systematic review and meta-analysis
Source: PLoS One. 2019 Jul 30;14(7):e0219888. doi: 10.1371/journal.pone.0219888 (PMC6667144; doi:10.1371/journal.pone.0219888)
Supplement: S5 Table — (PDF) [file pone.0219888.s005.pdf]

S4 Table. Risk of bias, by IDD

	Q1. Appropriate study design	Q2. Adequate sample size	Q3. Acceptable response rate	Q4. Reliable disability measure	Q5. Reliable outcome measure	Q6. Confounders analysed	Q7. Confidence intervals presented	Q8. Comparable cases and controls	Q9. Clearly defined cases and controls	Risk of Bias
**Autism**										
Almansour, 2013										Medium
Gong, 2015										Medium
Ingersoll, 2010										Medium
Lai, 2015										Medium
Riahi, 2010										High
Yang, 2015										Medium
**Cerebral Palsy**										
Altindag, 2007										Medium
Basaran, 2013										Medium
Cheshire, 2010										Medium
Kaya, 2010										Medium
Ones, 2005										Medium
Unsal-Delialioglu, 2009										Medium
Yilmaz, 2013										Medium
**Multiple: Combined**										
Cantwell, 2015										Medium
Norlin, 2013										Medium
Olsson, 2008										Medium
**Multiple: Disaggregated**										
Lach, 2009										Medium
Muammer, 2013										High
**Intellectual Disability**										
Gogoi, 2017										Medium
**Total**										
Low Risk	9 (47%)	15 (79%)	4 (21%)	14 (74%)	19 (100%)	0 (0%)	0 (0%)	11 (61%)	10 (56%)	0 (0%)
Medium Risk	10 (53%)	3 (16%)	2 (11%)	5 (26%)	0 (0%)	0 (0%)	19 (100%)	7 (39%)	8 (44%)	17 (89%)
High Risk	0 (0%)	1 (5%)	13 (68%)	0 (0%)	0 (0%)	19 (100%)	0 (0%)	0 (0%)	0 (0%)	2 (11%)
