# Supplementary material for: Depression and anxiety in parents of children with intellectual and developmental disabilities: A systematic review and meta-analysis
Source: PLoS One. 2019 Jul 30;14(7):e0219888. doi: 10.1371/journal.pone.0219888 (PMC6667144; doi:10.1371/journal.pone.0219888)

S1 Fig. Standardised mean difference in anxiety scores between parents of children with autism and the control group

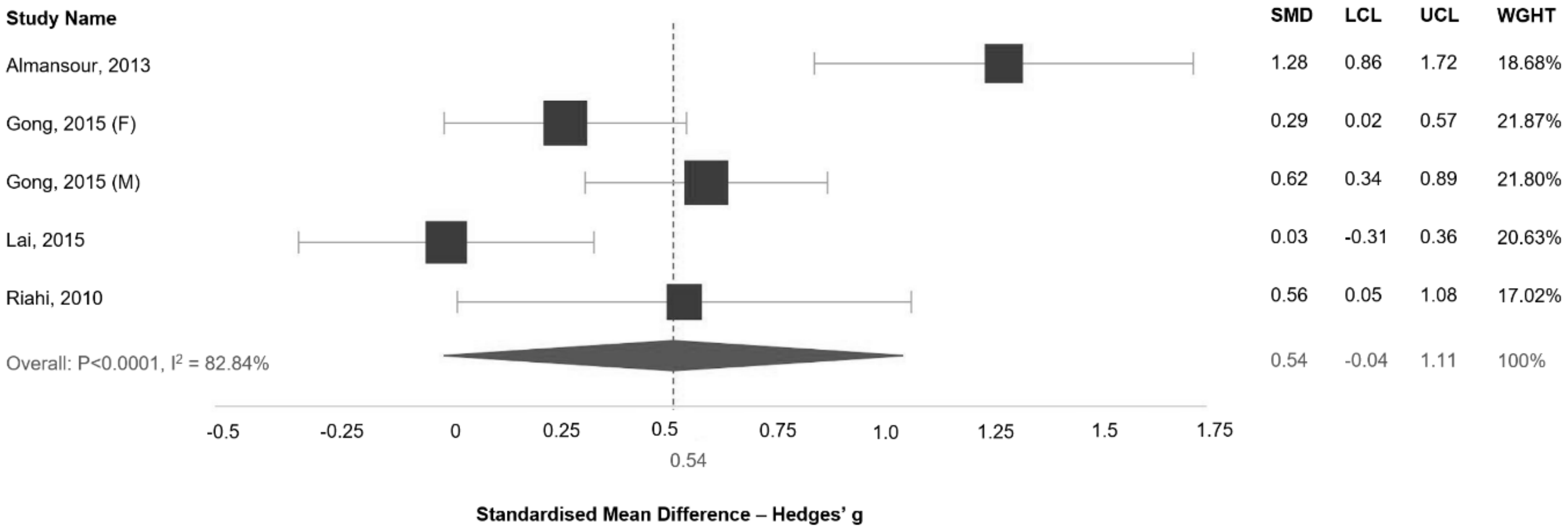

Supplement: S1 Fig — (PDF) [file pone.0219888.s006.pdf]
